# Clinical outcome of extremity arterial injuries in the modern era

**DOI:** 10.55730/1300-0144.6154

**Published:** 2026-01-25

**Authors:** Ali KUŞSAN, Selçuk COŞKUN, Alp ŞENER, Ferhat İÇME, Pınar KÖKSAL COŞKUN, Gülhan KURTOĞLU ÇELİK

**Affiliations:** 1 Department of Emergency Medicine, Ankara Bilkent City Hospital, Faculty of Medicine, University of Health Sciences, Ankara, Turkiye; 2 Department of Emergency Medicine, Faculty of Medicine, Yıldırım Beyazıt University, Ankara Bilkent City Hospital, Ankara, Turkiye; 3 Department of Cardiovascular Surgery, Ankara Etlik City Hospital, Faculty of Medicine, University of Health Sciences, Ankara, Turkiye

**Keywords:** Extremity arterial injury, computed tomography angiography, amputation, clinical presentation, upper extremity, lower extremity

## Abstract

**Background/aim:**

Extremity arterial injuries (EAIs) present a significant clinical challenge due to the risk of limb ischemia, amputation, and mortality. This retrospective cohort study aimed to delineate the clinical course, treatment strategies, and patient outcomes following trauma-induced EAIs, and to identify independent predictors of adverse outcomes.

**Materials and methods:**

Retrospectively analyzed were data from 168 consecutive patients with traumatic EAIs who underwent computed tomography angiography at a tertiary care center between 2019 and 2025. Data extracted from electronic medical records included demographics, injury mechanisms, clinical presentation, laboratory findings, imaging results, treatment modalities (primary repair, grafting, endovascular intervention, conservative management), and patient outcomes (sequelae-free recovery, amputation, mortality).

The primary outcome was sequelae-free recovery, while secondary outcomes included amputation, and mortality. The entire patient follow-up period, encompassing all treatment modifications, extended from emergency department admission until the primary or secondary outcome was reached. This period included hospitalization, postdischarge care (if applicable), and all subsequent outpatient clinic visits. Mortality was attributed only when directly causal.

**Results:**

The mean age of the patients was 37 ± 15 years, with a male predominance (86%). Penetrating trauma was the primary etiology for upper extremity injuries, while blunt trauma predominated in lower extremity injuries. Clinical presentation varied, with pulsatile bleeding significantly associated with penetrating trauma and hypoesthesia with blunt trauma. Independent predictors of adverse outcomes included hypotension, pulselessness, hypoesthesia, and elevated international normalized ratio (≥1.2). Base deficit was significantly associated with adverse outcomes in upper EAIs. Treatment modalities included primary repair (51.2%), grafting (10.1%), endovascular intervention (8.2%), and conservative management (28%). Amputation rates were 1.3% for upper extremities and 5.3% for lower ones, while mortality rates were 2.6% and 3.3%, respectively.

**Conclusions:**

Early diagnosis, prompt surgical intervention, and a multidisciplinary approach are essential for optimizing patient outcomes. Hypotension, pulselessness, and hypoesthesia were identified as significant independent predictors of adverse outcomes. Future multicenter studies are warranted to validate these findings.

## 1. Introduction

Extremity arterial injuries (EAIs), while relatively infrequent, represent a significant clinical challenge due to their potential for devastating consequences, including limb ischemia, amputation, and even mortality [1,2]. Prompt and accurate diagnosis is paramount for optimal patient management and the preservation of limb viability. Traditionally, conventional angiography has been the gold standard for evaluating vascular trauma. However, its invasive nature, associated risks, and time-consuming procedure have prompted the exploration of alternative imaging modalities. In recent years, computed tomography angiography (CTA) has emerged as a valuable tool in the assessment of EAIs [3]. Its noninvasive nature, rapid acquisition time, multiplanar reconstruction capabilities, and ability to simultaneously evaluate soft tissues and bony structures offer several advantages over conventional angiography.

This retrospective cohort study investigated the clinical course of trauma-induced EAIs diagnosed solely via emergency department (ED) CTA over a 5.5-year period. Demographic profiles, injury patterns, clinical and laboratory correlates, the impact of concomitant injuries, and treatment strategies were comprehensively evaluated to identify predictors of adverse outcomes, thereby aiming to refine the understanding of EAI management based on exclusive CTA diagnosis.

## 2. Materials and methods

### 2.1. Settings

This study was conducted at Ankara City Hospital, a tertiary care center in Türkiye with an annual ED volume exceeding 600,000 patient visits. Imaging was performed using a 128-slice multidetector computed tomography (CT) scanner (Revolution Evo 128, GE Healthcare, Milwaukee, WI, USA) allocated for ED use. The extremity CTA protocol was implemented in accordance with the manufacturer’s standardized instructions.

### 2.2. Study design

This retrospective cohort study included 1257 consecutive traumatic patients (pediatrics and adults) diagnosed with arterial injury (selected from a cohort of 168 patients who presented to our hospital’s ED with clinical findings suggestive arterial injury), between March 2019 and December 2024.

### 2.3. Inclusion and exclusion criteria

**Figure 1:**
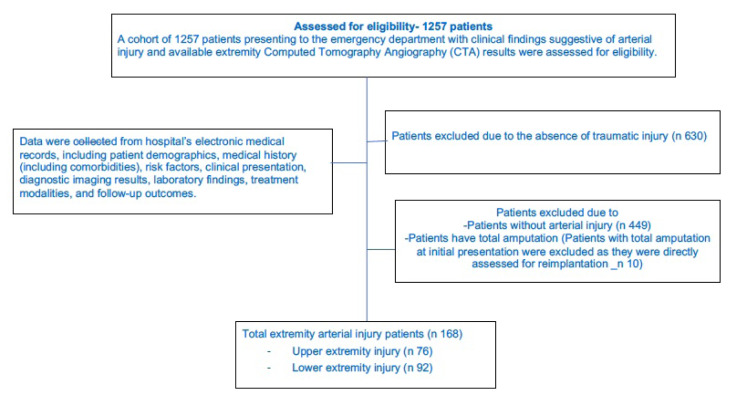
Flow diagram of the patients.

Patients whose data could not be accessed, who had incomplete data such as doctor’s notes or consultation notes, and who left the ED before completing their treatment were excluded from the study (shown in the flow diagram of the patients in Figure 1). Given that patients with total limb amputation at the time of initial evaluation were immediately assessed for reimplantation, they were not included in this analysis of outcomes following CTA.

### 2.4. Data collection

Data were collected from electronic medical records, and included patient demographics, medical history (including comorbidities), risk factors, clinical presentation, diagnostic imaging results, laboratory findings, treatment modalities, and follow-up outcomes. Clinical data were abstracted by the researchers using a standardized data collection form.

### 2.5. Outcome measures

The primary outcome was sequelae-free recovery, while the secondary outcomes included amputation and mortality. The entire patient follow-up period, encompassing all treatment modifications, extended from ED admission until the primary or secondary outcome was reached. This period included hospitalization, postdischarge care (if applicable), and all subsequent outpatient clinic visits. Mortality was attributed only when directly causal; mortality associated with concomitant injuries was excluded from the reported mortality.

Abbreviated Injury Scale (AIS) and Injury Severity Score (ISS) scores were not systematically recorded for all patients due to inconsistencies in historical records. This important limitation prevented standardized assessment and a comparison of injury severity.

Since the numbers of cases for mortality and amputation outcomes were individually very low, these were combined under a new composite outcome termed poor outcome (defined as mortality or amputation), and binary logistic regression analysis was conducted based on this combined variable.

### 2.6. Ethical considerations

Ethical approval was obtained from the institutional review board of Hospital (TABED 2-24-117) prior to study commencement. All research procedures were conducted in accordance with the Declaration of Helsinki.

### 2.7. Timeline

The index date was defined as the date of the initial ED presentation. Specific injured arteries were identified from formal radiology reports of ED CTA. Initial treatment regimens and subsequent modifications during follow-up were recorded. Surgical interventions were categorized as surgical procedures, and angiographic interventions were categorized as interventional procedures. Blood transfusions were documented throughout the entire hospitalization period.

### 2.8. Statistical analysis

Statistical analyses were performed using IBM SPSS Statistics for Windows 20.0 (IBM Corp., Armonk, NY, USA). Continuous variables with normal distribution were expressed as the mean ± standard deviation (paired t test). Categorical variables were compared using Pearson’s chi-squared test. For the poor outcome variable, all covariates demonstrating significance at p < 0.250 in the primary analysis were included in the binary logistic regression model. The independent predictors were presented with odds ratios (ORs) and 95% confidence intervals (CIs). Statistical significance was accepted as p < 0.05. Patients with inaccessible data (n = 40) were excluded from the study, and statistical analyses were performed on the remaining cohort.

## 3. Results

A total of 168 consecutive patients with acute traumatic arterial injury were included. Patient demographics (age and sex), etiological mechanism, local examination findings at ED presentation, affected artery, concomitant injuries, initial laboratory results, administered treatment, amputation rates, and mortality outcomes are summarized in Table 1, which also presents a statistical comparison between upper and lower EAIs, as indicated by the highlighted section.

Although etiological evaluation traditionally focuses on blunt and penetrating trauma, this patient group exhibited a notably high incidence of firearm injuries/gunshot wounds. Therefore, it was deemed appropriate to categorize the analysis into three groups: blunt trauma, sharp penetrating trauma, and firearm injuries. Tables 2 and 3 present the affected arteries and initial physical examination findings according to these etiological mechanisms.

Pearson’s chi-squared test results revealed statistically significant associations between the initial local examination findings and specific injury etiologies across the three study groups. Specifically, pulsatile bleeding was significantly associated with the sharp penetrating injury group, while hypoesthesia was significantly associated with the blunt trauma group. Regarding the injured artery, sharp penetrating trauma was identified as a significant etiological factor in upper EAIs, whereas blunt trauma was significantly associated with lower EAIs. Furthermore, the analysis of concomitant injuries demonstrated a higher prevalence of nerve injuries in the sharp penetrating trauma group, and a higher prevalence of bone fractures in the blunt trauma group.

**Figure 2:**
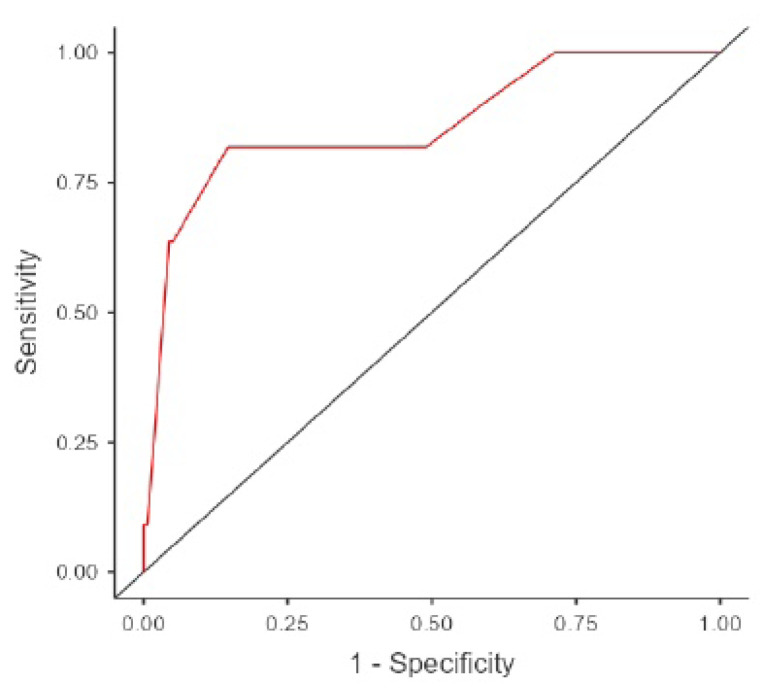
ROC curve of the multiple logistic regression model.

To identify predictors of mortality and major amputation, comparative analyses were performed between patient groups stratified by outcome, encompassing age, comorbidities (diabetes mellitus, hypertension, coronary artery disease), clinical findings (pulsatile bleeding, pulselessness, hypoesthesia, hematoma), laboratory values (hemoglobin, platelet count, international normalized ratio (INR), base excess (BE), expressed as base deficit (BD) when negative, lactate, white blood cell count, neutrophil, monocyte, and lymphocyte counts, neutrophil/lymphocyte ratio), injury characteristics (proximal/distal location, concomitant nerve, venous, and bone injuries), and red blood cell transfusion requirements. This identified hypotension, pulselessness, hypoesthesia, and elevated INR as independent predictors of mortality and major amputation. Receiver operating characteristic (ROC) analysis, using the Youden index, determined an optimal INR cutoff of ≥1.2. The INR variable was then dichotomized based on this cutoff, and the multiple logistic regression was repeated. These findings are detailed in Table 4 and Figure 2 (ROC curve of the multiple logistic regression model).

BD emerged as a critical factor in the analysis of mortality and major amputation. In upper extremity arterial trauma, a statistically significant difference in the mean BD was observed, with patients experiencing mortality and major amputation exhibiting a substantially lower mean BD (−7.2 ± 1.8) compared to those without (−2.4 ± 2.7, p = 0.007). Conversely, in lower extremity arterial trauma, while a trend toward lower mean BD was noted in patients with mortality and major amputation (−5.7 ± 7.3 vs. −4.2 ± 5.3), this difference did not reach statistical significance (p = 0.656).

## 4. Discussion

EAIs present a critical challenge in trauma care, requiring prompt diagnosis and intervention to prevent limb loss and life-threatening sequelae. Frequently resulting from high-energy trauma, such as motor vehicle collisions or penetrating injuries, EAIs necessitate rapid vascular assessment to guide treatment and optimize outcomes. Delays in diagnosis and management can lead to irreversible ischemia, potentially culminating in amputation, significant morbidity, and even mortality [2]. CTA, with its noninvasive, rapid, and comprehensive visualization capabilities, has become the preferred modality for evaluating EAIs, providing unparalleled detail for informed clinical decision-making. Its noninvasive nature, rapid acquisition, and capacity to visualize both osseous and soft tissues provide unparalleled detail regarding injury localization, vessel wall integrity, and collateral flow, empowering informed clinical decision-making [3,4]. This retrospective study of 168 trauma patients, presenting with EAIs from blunt, penetrating, or gunshot injuries, utilized CTA data from admission to final outcomes, spanning the hospital’s records. Epidemiological studies consistently demonstrate age and sex disparities in trauma incidence, with younger individuals and males disproportionately affected [5–7]. In this present cohort, the mean age was 37 ± 15 years, confirming this trend. Furthermore, a significant male predominance was observed, with 86% of injuries occurring in males, aligning with established literature. This sex disparity is likely attributable to increased occupational risks and engagement in high-risk activities among men. The mean age findings herein are congruent with those of previous reports, reflecting the elevated trauma risk in younger, active populations. Regarding injury mechanisms, penetrating and blunt trauma were the predominant etiologies of EAIs. While these findings are consistent with general trauma literature, regional variations in violence rates and occupational hazards may influence injury patterns, necessitating context-specific interpretations.

Trauma mechanisms for arterial injuries vary across studies. While Franz et al. [8,9] found gunshot wounds dominant in lower extremity injuries and Tan et al. [6] reported blunt trauma as the leading cause overall, the current study revealed distinct patterns: penetrating trauma predominates in upper extremity injuries (71.1%), whereas blunt trauma leads in lower extremity injuries (51.1%). These discrepancies likely reflect differences in the study populations and settings. Speculations exist regarding trauma mechanisms. Lower extremities, due to anatomical position and function, are more prone to blunt trauma from accidents and falls. Upper extremities are more vulnerable to penetrating trauma from sharp objects and projectiles. Occupational hazards further influence injury patterns; construction workers face higher risks of lower extremity blunt trauma, while those in high-crime areas or handling sharp objects are more susceptible to upper extremity penetrating injuries.

Clinical presentation and diagnosis: The clinical presentation of patients with EAIs in the present cohort exhibited a spectrum of hemorrhagic and ischemic manifestations, including pulsatile bleeding, hematoma, pulselessness, and hypoesthesia. Notably, the presence of pulselessness, hypoesthesia, and hematoma demonstrated a significant association with bad patient outcomes, emphasizing the critical importance of early diagnosis and prompt intervention in this patient population. Upon hospital admission, trauma patient symptom presentations revealed a disparity between our findings and those reported by Gallo et al. [10], who documented a 76.1% hemorrhage and 23.9% ischemia distribution. In the current cohort, hemorrhage (pulsatile bleeding, hematoma) was observed in 64.8% of patients, while ischemia (pulselessness, hypoesthesia) was present in 76.1%. These deviations may stem from regional variations, differences in symptom classification, and the frequent cooccurrence of hemorrhagic and ischemic symptoms in the patient population. Furthermore, our institution’s role as a regional referral center for invasive procedures likely contributes to a higher incidence of ischemic presentations, as patients from distant locations are frequently transferred. The influence of prolonged transport on ischemia development warrants further investigation.

Beyond limb viability, EAIs can trigger systemic consequences. Ischemia and blood loss can induce metabolic acidosis, reflected in elevated BD levels [11,12]. EAIs can induce systemic metabolic acidosis, reflected by elevated BD. BD, a marker of tissue hypoperfusion, correlates with poor outcomes in trauma. In EAIs, ischemia severity likely influences BD, though the precise relationship with CTA-defined injury remains unclear. The analysis herein revealed a significant association between BD and mortality and major amputation in upper EAIs. Patients with mortality and major amputation exhibited a significantly lower mean BD (−7.2 ± 1.8) compared to those without (−2.4 ± 2.7, p = 0.007). In lower EAIs, while a trend toward lower BD in mortality and major amputation (−5.7 ± 7.3 vs. −4.2 ± 5.3) was observed, it did not reach statistical significance (p = 0.656).

Furthermore, the presence of associated injuries, such as nerve and bone injuries, significantly impacted patient outcomes [1,13–15]. The current investigation revealed distinct patterns of concomitant injuries in upper and lower extremity arterial trauma. These findings underscore the imperative of a comprehensive evaluation of patients with EAIs to identify and address all associated injuries for optimal patient management. In upper extremity injuries, nerve injuries were observed in a notable 51.3% of cases, with bone fractures in 23.7% and venous injuries in 1.3%. Conversely, lower extremity injuries were characterized by a predominance of bone fractures (58.7%), followed by venous injuries (10.9%), and a low rate of nerve injuries (1.1%). While prior literature has not extensively examined the impact of these concomitant injuries on amputation or mortality, this study established a significant association between bone fractures accompanying upper EAIs and mortality and major amputation. Similarly, venous injuries in lower extremity arterial trauma were identified as a significant predictor of mortality and major amputation.

The findings herein suggest a potential trend: upper EAIs appear to frequently involve nerve injuries, and the presence of bone fractures may significantly elevate the risk of adverse outcomes. Conversely, lower extremity injuries seem to be more commonly associated with bone fractures, and the presence of venous injuries and hematomas may contribute to less favorable outcomes. While further research is needed to confirm these observations, our study supports this potential relationship between injury location, associated injuries, and patient outcomes.

Our study suggests a possible link between elevated BD and adverse outcomes in patients with upper EAIs, which is consistent with previous research indicating the prognostic value of BD in critically ill patients. It was also observed that the need for blood transfusion was notably higher in patients with lower extremity injuries, likely due to increased blood loss. In these cases, the initial severe hemorrhage may lead to a higher demand for red blood cells. Subsequently, hematoma formation could provide a tamponade effect, potentially limiting further blood loss and thus reducing the development of significant metabolic acidosis, which would be reflected in a lower BD. In contrast, the absence of this buffering effect in upper extremity injuries might make BD a more sensitive prognostic marker in this patient group. While these findings warrant further investigation to fully understand the prognostic significance of BD and its potential clinical utility, this study supports the observed association between elevated BD and adverse outcomes in upper EAIs.

Elevated INR emerged as a significant predictor of adverse outcomes in our EAI cohort, suggesting that preexisting coagulopathies, the systemic response to severe injury potentially manifesting as trauma-induced coagulopathy, or underlying liver dysfunction may independently contribute to poorer outcomes beyond the local vascular damage. This finding underscores the importance of considering the coagulation profiles and systemic status of patients alongside the arterial injury itself when assessing risk and planning management.

Treatment strategies and outcomes: Treatment modalities varied based on the severity of the injury and the presence of associated comorbidities [16–18]. Surgical intervention constituted the mainstay of treatment for the majority of patients, with primary repair being the most frequently employed procedure. Notably, endovascular interventions have emerged as a substantial option within the realm of interventional radiology, demonstrating promising results in clinical trials. This trend indicates its potential to become a pivotal therapeutic modality in future clinical practice, particularly for the treatment of complex and challenging conditions.

Clinical implications:

- Early diagnosis and intervention: prompt diagnosis and aggressive management are crucial for optimal outcomes.
- Multidisciplinary approach: a multidisciplinary approach involving emergency physician, trauma surgeons, vascular surgeons, intensivists, and other specialists is essential for optimal patient care.
- Risk stratification: developing clinical prediction rules to identify patients at high risk for mortality and major amputation may facilitate early intervention and improve patient outcomes [19–21].

This study revealed the following treatment distribution: 51.2% primary repair, 10.1% graft treatment, 8.2% endovascular intervention, 28% conventional management, and 2.4% extremity amputation. Notably, a significant proportion (78.7%) of patients managed conventionally sustained lower extremity injuries. Comparative analysis with published mortality and major amputation rates suggests that conventional management did not significantly impact patient outcomes. Further investigation is warranted to rigorously evaluate the comparative efficacy of conventional and surgical treatment strategies. In patients presenting with gunshot injuries, a higher prevalence of surgical interventions was observed: 37.9% primary repair, 27.6% graft treatment, and 6.9% endovascular intervention. This suggests a propensity for clinicians to favor conventional management in lower extremity blunt trauma and operative interventions in gunshot injuries.

Amputation rates were consistent with the literature. Gallo et al. [10] reported 1.3% amputation for upper EAIs and 5.7% for lower ones. Franz et al. [8,9] noted upper and lower extremity amputation rates of 0.7% and 4.8%, respectively. Sciarretta et al. [14] reported a 17% amputation rate in popliteal artery injuries. The present study showed upper and lower extremity amputation rates of 1.3% and 5.3%, respectively, aligning with these findings.

Gallo et al. [10] found 30-day mortality rates of 2.1% for upper EAIs and 3.3% for lower ones. Kim et al. [23] reported a 2% mortality rate for upper EAIs and 6% for lower ones. The mortality rates in the current study were 2.6% for upper extremity injuries and 3.3% for lower ones, consistent with existing literature. Multiple logistic regression analysis of mortality and major amputation, following INR categorization (Table 4), identified hypotension, pulselessness, hypoesthesia, and elevated INR as significant predictors. These findings require validation through further studies.

In terms of limitations and future directions, while acknowledging the inherent limitations stemming from this study’s retrospective design and the resultant data omissions—specifically regarding crucial clinical and imaging variables such as the interval before hospital presentation, the differentiation between pseudoaneurysm and occlusion, restraint usage in blunt trauma cases, tourniquet duration, the type of vascular bypass (autologous versus prosthetic), initial blood pressure readings, toxicology screening results, cooccurring injuries, and preexisting medical conditions—this analysis still provides significant and noteworthy insights into the studied phenomenon.

To address the need for a modern era perspective and strengthen literature integration, our findings align with recent metaanalyses and systematic reviews, enhancing the understanding of EAI management. Qi et al. [22] conducted a metaanalysis of 32 studies, encompassing 863 patients with vascular trauma, and reported that early intervention within 6 h significantly reduces amputation rates (OR: 0.42, 95% CI: 0.21–0.85) and mortality (OR: 1.11, 95% CI: 0.75–1.64), emphasizing the critical role of rapid diagnosis and treatment. Similarly, Kim et al. [1] performed a nationwide analysis and found endovascular approaches associated with lower morbidity and mortality (5.5% vs. 14.8% p = 0.045), reduced blood loss, and shorter hospital stay [23]. The outcomes herein, with low amputation (1.3% upper, 5.3% lower extremity) and mortality (2.6% upper, 3.3% lower) rates, corroborate these findings, as our use of CTA facilitated timely interventions, with endovascular procedures (8.2%) proving effective, especially for penetrating injuries. Comparing our risk stratification predictors—hypotension, pulselessness, hypoesthesia, and elevated INR (≥1.2)—with established scoring systems like the Mangled Extremity Severity Score (MESS), Rutherford classification, and Mangled Extremity Severity Index (MESI) provides further context. MESS, which assigns points for ischemia duration, shock, age, and injury energy, predicts amputation risk with scores ≥7 [24]. The predictors used in this study align with MESS components like shock (hypotension) and ischemia (pulselessness, hypoesthesia), though the retrospective absence of systematic AIS/ISS data limited direct MESS application. The Rutherford classification, widely used for acute limb ischemia, categorizes severity based on sensory loss, motor deficits, and Doppler signals, with categories IIb and III indicating urgent intervention [25]. Our findings of hypoesthesia and pulselessness correspond to these higher-risk categories, reinforcing their prognostic value. The older MESI, incorporating similar clinical variables, is less specific but still relevant [26]. Notably, our identification of elevated INR as a novel predictor suggests potential enhancements to these scoring systems, as coagulopathy may exacerbate outcomes independently of local vascular injury. These insights, supported by recent literature, highlight the importance of early CTA-driven diagnosis, evolving endovascular techniques, and refined risk stratification in optimizing EAI outcomes in the modern era.

This study had several limitations, including its retrospective design, single-center nature, and potential for selection bias. Additionally, the relatively small sample size and single-center design may limit the generalizability of the findings. Future studies with larger sample sizes and multicenter collaborations are needed to further validate these findings and investigate novel therapeutic strategies.

In conclusion, EAIs require rapid, decisive action. The current analysis underscores the critical role of early diagnosis, timely surgical intervention, and a coordinated multidisciplinary approach in optimizing patient outcomes. Hypotension, pulselessness, hypoesthesia, and elevated INR emerged as key predictors of adverse outcomes, highlighting the need for robust risk stratification tools. Future multicenter studies, leveraging larger cohorts, are essential to validate these findings, refine clinical prediction rules, and explore novel therapeutic strategies aimed at improving patient outcomes in these complex injuries.

## Figures and Tables

**Table 1:** Demographics of the patients

	Total	Upper Extremity	Lower Extremity	p value

	168	76	92	

**Age (years)**	37 (11–81)	37 ± 14	37 ± 16	0.533

**Male**	145	66	79	0.855

**Affected Arteries**		Axillary 1 (0.6%)	Femoral 25 (14.9 %)	
		Brachial 6 (3.6%)	Popliteal 18 (10.7%)	
		Radial 41 (24.4%)	Ant tibial 33 (19.6%)	
		Ulnar 29 (17.3)	Peroneal 17 (10.1%)	
		Other 3 (1.8%)	Post tibial 2 (%1.2)	
			Other 7 (4.2%)	

**Mechanism**				
**Blunt (n, age)**	64 (38.1%) 39±17 years	17 (22.4%)	47 (51.1%)	<0.001
**Penetrating (n, age)**	75 (44.6%) 35±15 years	54 (71.1%)	21 (22.8%)	<0.001
**Gunshot (n, age)**	29 (17.3%) 35±10 years	5 (6.6%)	24 (26.1%)	<0.001

**Examination Findings**				
**Pulsatile Bleeding**	42 (25%)	25 (32.9%)	17 (18.75%)	0.032
**Pulselessness**	54 (32.1%)	24 (31.6%)	30 (32.6%)	0.887
**Hypoesthesia**	74 (44%)	38 (50%)	36 (39.1%)	0.158
**Hematoma**	67 (39.9%)	24 (31.6%)	43 (%46.7%)	0.046

**Accompanying Injuries**				
**Nerve injury**	40 (23.8%)	39 (51.3%)	1 (1.1%)	<0.001
**Vein injury**	11 (6.5%)	1 (1.3%)	10 (10.9%)	<0.001
**Bone fracture**	72 (42.9%)	18 (23.7%)	54 (58.7)	0.013
				
**Laboratory Findings**				
**Hematocrit**	39.3±6.6	39.9±5.7	38.7±7.2	0.265
**Leukocyte**	13.87±6.74	11.5±4.4	15.82±7.67	<0.001
**Platelets**	263000±74000	247±63	276±81	0.026
**Lactate**	3.45±2.61	2.60±1.15	3.94±3.06	0.015
**Base excess**	−3.8±4.7	−2.7±2.9	−4.4±5.5	0.128
**Neutrophil**	10.49±6.47	8.32±4.54	12.28±7.26	<0.001
**Monocyte**	0.63±0.32	0.50±0.17	0.74±0.37	<0.001
**Lymphocyte**	2.47±1.5	2.40±1.32	2.53±1.63	0.943
**Neutrophil/Lymphocyte**	6.44±6.59	5.48±5.87	7.24±7.06	0.028

**Treatment**				
**Blood transfusion**	44 (26.2%)	11 (14.5%)	33 (35.9%)	0.002
**Operation (total)**	107 (63.7%)	63 (82.9%)	44 (47.8%)	<0.001
**Primary Repair**	86 (51.2%)	57 (75%)	29 (31.5%)	<0.001
**Graft Repair**	17 (10.1%)	5 (6.6%)	12 (13.0%)	<0.001
**Amputation (initial)**	4 (2.4%)	1 (1.3%)	3 (3.3%)	<0.001
**Endovascular procedure**	14 (8.3%)	3 (3.9%)	11 (12%)	<0.001
**Conventional Follow up**	47 (28%)	10 (13.2%)	37 (40.2%)	<0.001

**Amputation at the end of the treatment process**	6 (3.6%)	1 (1.3%)	5 (5.4%)	0.223

**Mortality (Emergency dept/Hospital)**	5 {1(0.6%)/5(3%)}	2 {0 (0%)/2 (2.6)}	3 {1 (1.1%)/3 (3.3%)}	<0.001/ 1

**Poor outcome (amputation +mortality)**	11 (6.5 %)	3 (3.9%)	8 (8.7%)	0.348

**Table 2:** Statistical evaluation of the association between injury mechanisms and arterial injury.

	Blunt (n /%)	Sharp penetrating (n, %)	Gunshot wound (n, %)	p value
**Male**	48/75.0%	69/92.0%	28/96.6%	0.003
**Pulsatile Bleeding**	6/9.4%	30/40.0%	6/20.7%	<0.001
**Pulselessness**	25/39.1%	21/28.0%	8/27.6%	0.321
**Hypoesthesia**	39/60.9%	25/33.3%	10/34.5%	0.003
**Hematoma**	24/37.5%	28/37.3%	15/51.7%	0.359

**Table 3:** Association between the trauma mechanism and injured structures.

	Blunt	Sharp penetrating	Gunshot wound	p value
**Upper Extremity-Total**	17 (22.4%)	54 (71.1%)	5 (6.6%)	<0.001
**Axillary Artery**	0 (0.0%)	0 (0.0%)	1(100.0%)	-
**Brachial Artery**	1 (16.7%)	5 (83.3%)	0 (0.0%)	-
**Radial Artery**	12 (29.3%)	27 (65.9%)	2 (4.9%)	0.003
**Ulnar Artery**	7 (24.1%)	22 (75.9%)	0 (0.0%)	<0.001
**Other Upper Arteries**	0 (0.0%)	3 (100.0%)	0 (0.0%)	-
**Lower Extremity-Total**	47 (51.1%)	21 (22.8%)	24 (26.1%)	<0.001
**Femoral Artery**	10 (40.0%)	9 (36.0%)	6 (24.0%)	0.524
**Popliteal Artery**	6 (33.3%)	4 (22.2%)	8 (44.4%)	0.004
Tibial Artery	19 (57.6%)	5 (15.2%)	9 (27.3%)	0.001
Peroneal Artery	16 (94.1%)	1 (5.9%)	0 (0.0%)	<0.001
Posttibial Artery	2 (100.0%)	0 (0.0%)	0 (0.0%)	-
Other Lower Arteries	2 (28.6%)	2 (28.6%)	3 (42.9%)	-
Proximal/Distal	17 (32.7%)/47 (40.5%)	17(32.7%)/58 (50.0%)	18 (34.6%)/11 (9.5%)	<0.001
Nerve Injury	6 (15%)	33 (82.5%)	1 (2.5%)	<0.001
Vein Injury	3 (27.3%)	4 (36.4%)	4 (36.4%)	-
Bone Fracture	52 (72.2%)	6 (8.3%)	14 (19.4%)	<0.001

Pearson chi-squared test results.

**Table 4:** Multiple logistic regression analysis of mortality and major amputation conducted following INR categorization.

	B	p value	Odds Ratio (CI 95%)
**Hypotension**	2.828	0.012	16.919 (1.88–152.235)
**Pulselessness**	2.042	0.012	7.704 (1.558–38.097)
**Hypoesthesia**	2.201	0.019	9.031 (1.442–56.571)
**Elevated INR**	1.604	0.026	4.975 (1.213–20.399)
Variables entered: Hypotension, Pulselessness, Hypoesthesia, Elevated INR. Model diagnostic statistics: AUC 0.935, Accuracy 0.935.			

Area under the ROC curve was 0.935. As with the ROC curve, an AUC of 0.935 indicates quite remarkable discrimination. This means the model had a very high ability to correctly distinguish between the two outcome groups. The accuracy of the model was 0.935, or 93.5%. This indicates that the model correctly classified 93.5% of the cases in the dataset.

## Data Availability

Data and materials are available through hospital automation information systems.
